# Identification of a novel fungus, *Trichoderma asperellum* GDFS1009, and comprehensive evaluation of its biocontrol efficacy

**DOI:** 10.1371/journal.pone.0179957

**Published:** 2017-06-23

**Authors:** Qiong Wu, Ruiyan Sun, Mi Ni, Jia Yu, Yaqian Li, Chuanjin Yu, Kai Dou, Jianhong Ren, Jie Chen

**Affiliations:** 1 Department of Environment and Resource, School of Agriculture and Biology, Shanghai Jiao Tong University, Shanghai, China; 2 State Key Laboratory of Microbial Metabolism, Shanghai Jiao Tong University, Shanghai, China; 3 The Key laboratory of Urban (South) Agriculture, Ministry of Agriculture, Shanghai, China; 4 State Key Laboratory of Plant Genomics and National Center for Plant Gene Research (Beijing), Institute of Genetics and Developmental Biology, Chinese Academy of Sciences, Beijing, China; 5 Suzhou BioNovoGene Metabolomics Platform, Suzhou, China; Universita degli Studi di Pisa, ITALY

## Abstract

Due to its efficient broad-spectrum antimicrobial activity, *Trichoderma* has been established as an internationally recognized biocontrol fungus. In this study, we found and identified a novel strain of *Trichoderma asperellum*, named GDFS1009. The mycelium of *T*. *asperellum* GDFS1009 exhibits a high growth rate, high sporulation capacity, and strong inhibitory effects against pathogens that cause cucumber fusarium wilt and corn stalk rot. *T*. *asperellum* GDFS1009 secretes chitinase, glucanase, and protease, which can degrade the cell walls of fungi and contribute to mycoparasitism. The secreted xylanases are good candidates for inducing plant resistance and enhancing plant immunity against pathogens. RNA sequencing (RNA-seq) and gas chromatography-mass spectrometry (GC-MS) showed that *T*. *asperellum* GDFS1009 produces primary metabolites that are precursors of antimicrobial compounds; it also produces a variety of antimicrobial secondary metabolites, including polyketides and alkanes. In addition, this study speculated the presence of six antimicrobial peptides via ultra-performance liquid chromatography quadrupole time of flight mass spectrometry (UPLC-QTOF-MS/MS). Future studies should focus on these antimicrobial metabolites for facilitating widespread application in the field of agricultural bio-control.

## Introduction

*Trichoderma* spp. exhibits antagonistic effects against at least 18 genera and 29 species of pathogenic fungi, as well as a variety of pathogenic bacteria. The biocontrol mechanisms of *Trichoderma* spp. primarily include competition and mycoparasitism, followed by the stimulation of plant resistance and immunity [1].

*Trichoderma* spp. is highly adaptive to the environment, and their growth rates are generally much faster than those of plant pathogens. *Trichoderma* spp. could compete with nearby pathogens for limited space and nutrients, thereby inhibiting their growth. Competition mechanism plays an important role in the inhibition of soil-borne diseases and leaf pathogens [2, 3].

The most significant antagonistic mechanism of *Trichoderma* spp. against pathogens is mycoparasitism. After recognizing the pathogen, the *Trichoderma* mycelium grows alongside the pathogen mycelium in a spiral fashion, resulting in dissolution and death of the pathogen. This process is accompanied by the secretion of cell wall-degrading enzymes (CWDEs), such as chitinases, glucanase, and proteases, which penetrate the pathogen mycelium, absorbing its nutrients and ultimately dissolving the pathogen [4–6]. As is well known, the chitinase protein CHIT33 from *Trichoderma harzianum* CECT2413 exerts a significant inhibitory effect on *Fusarium* [7]. Overexpression of the endochitinase gene (*chit36*) from *T*. *harzianum* Rifai TM results in complete inhibition of *Botrytis cinerea* spore germination [8]. The alkaline protease gene *prb1*, isolated from *T*. *harzianum*, is associated with mycoparasitism [9]. Exo and endo-β-1, 3-glucanase are also involved in the mycoparasitism of *Trichoderma* [10, 11].

When interacting with plants, *Trichoderma* spp. produce elicitors, which induce systemic resistance in plants and enhance their immunity against pathogens. The xylanase protein XYN2 secreted by *T*. *reesei* QM6a causes tissue necrosis and induces systemic resistance in tobacco by stimulating the ethylene signaling pathway and regulating the overexpression of downstream resistance-associated proteins such as PR2b and ERF2 [12]. SM1, a small 14-kDa protein secreted by *T*. *viride* Gv 29–8, induces immune resistance in plants by stimulating the ethylene-jasmonic acid signaling pathway [13, 14]. In addition, several other elicitors have been discovered and found to induce plant systemic resistance [15].

*Trichoderma* spp. induces pathogen inhibition by secreting secondary metabolites. Different *Trichoderma* species secrete different substances, including isonitrile, diketopiperazines, sesquiterpenes, stemids, polyketides, alkylpyrones, and peptaibols [16]. It has been shown that 6-pentyl-α-pyrone, produced by *T*. *harzianum*, efficiently inhibits the growth of *Rhizoctonia solani* on rice [17]. At present, more than 300 compounds have been identified as antimicrobial peptaibols, including 11 from *T*. *longibrachiatum* and one peptaibol polymer with 20 peptaibol residues from *T*. *brevicompactum* [18, 19].

Previously, research of *Trichoderma* spp. mainly focused on only one or a few biocontrol factors in one strain. Our study reveals the resistance-related molecular mechanisms of *T*. *asperellum* GDFS1009, including almost all of the biocontrol factors related to mycoparasitism, induced resistance, and antibiosis, using genomics, transcriptomics, and metabolomics approaches, as well as a series of physiological and biochemical analyses. These data provide a good foundation for continued researches into *T*. *asperellum* GDFS1009 for facilitating widespread application in the field of agricultural bio-control.

## Materials and methods

### Isolation and morphological and molecular identification of a novel strain

Irrigated soil sample was collected in Foshan, Guangdong, China via the diagonal method. No specific permissions were required for the location and the related activities about the soil sample, because the sample was collected on private land and we confirm that the owner of the land gave permission to conduct the study on this site. Furthermore, we confirm that the field studies did not involve endangered or protected species.

Via dilution plating, rose bengal-agar culture plates containing 50 μg/mL streptomycin and 50 μg/mL chloramphenicol were used to isolate *Trichoderma* spp., among which a novel strain *T*. *asperellum*, named GDFS1009, was discovered by morphological observation. Then the classification position was confirmed by ITS sequencing.

*T*. *asperellum* GDFS1009 was cultured in potato-dextrose (PD) broth in a rotary shaker for 2 d at 28°C and 180 rpm until its mycelium was well developed. The supernatant was removed by vacuum filtration, the mycelium was collected as the pellet, and the genome was extracted using the hexadecyl trimethyl ammonium bromide (CTAB) method [20]. Using universal ITS primers (ITS1-F and ITS4) [21], ITS sequences were amplified by PCR using the *T*. *asperellum* GDFS1009 genome as the template. The PCR product was ligated into the pMD19-T vector (Takara, Shiga, Japan) and was then sequenced by the Shanghai Biosune Biotechnology Co. (Shanghai, China). The sequencing results were aligned with the ITS sequences of known strains using NCBI BLAST (http://www.ncbi.nlm.nih.gov/BLAST). MEGA 4.0 software was used to build a phylogenetic tree for ITS [22].

### Detection of biocontrol efficacy

Discs were obtained from 4-day-old cultures of pathogens (*Fusarium oxysporum* f. sp. *cucumerinum* Owen and *Fusarium graminearum*) and *T*. *asperellum* GDFS1009 using sterile 7 mm hole-punchers. Each disc was inverted and placed on one side of a 9-cm Petri dish with PDA medium, with 4 cm between pathogen and *T*. *asperellum* GDFS1009 discs. The control was a dish containing only the pathogen disc. Dishes were incubated at 28°C for 5 d [23].

Then spore suspension (10^6^ cfu/mL) of *T*. *asperellum* GDFS1009 was cultured in PD broth in a rotary shaker at 28°C and 180 rpm for 6 d. A total of 50 mL of filtered fermentation liquor from *T*. *asperellum* GDFS1009 or ddH_2_O (control) were mixed into 100mL PDA medium, and the plate was made in 9-cm Petri dish. 7 mm discs of 4-day-old cultures of pathogens (*Fusarium oxysporum* f. sp. *cucumerinum* Owen and *Fusarium graminearum*) were inverted and placed on the center of each plate, then were incubated at 28°C for 4 d. Additionally, related micro well dilution assay of pathogens inhibition was also done with filtered fermentation liquor from *T*. *asperellum* GDFS1009.

Cucumber (Jinyan no. 4) plants were grown to the 3–5-leaf stage in soil under a 10-h light/14-h dark cycle in a 28°C phytotron. A 20-mL spore suspension of *F*. *oxysporum* f. sp. *cucumerinum* Owen (10^6^ cfu/mL) was irrigated onto the roots of cucumber seedlings, followed by a 20-mL spore suspension of *T*. *asperellum* GDFS1009 (10^7^ cfu/mL) in the treatment group. The control group was irrigated with sterile water instead of *T*. *asperellum* GDFS1009. The inoculated plants were placed in the 28°C phytotron for at least 7 d, and biocontrol effects were observed and analyzed [24].

### Assay of enzymes related to mycoparasitism and induced resistance

*T*. *asperellum* GDFS1009 was cultured in PD broth in a rotary shaker at 28°C and 180 rpm for 2 d until the mycelium was well developed. The supernatant was removed by vacuum filtration, and 1 g of mycelium pellet was transferred to xylanase induction medium includes 2% corncob powder, 0.4% NH4NO3, 0.05% K2HPO4, 0.05% MgSO4·7H2O, 0.05% KCl, and 0.001% FeSO4·7H2O. The activities of xylanase were measured using 3, 5-dinitrosalicylic acid (DNS). At 50°C and pH 5.0, 1 U of xylanase activity was defined as the amount of enzyme required to release 1 μmol xylose per minute. Xylose was used as standard for xylanase analysis [25].

Then spore suspension (10^4^ cfu/mL) of *T*. *asperellum* GDFS1009 was cultured in protease induction medium (1% casein, 0.05% K2HPO4, 0.05% MgSO4·7H2O, 0.05% KCl, and 0.001% FeSO4·7H2O) in a rotary shaker at 28°C and 180 rpm for 5 d. The activities of protease were measured every day using foline-phenol reagent. At 50°C and pH 7.5, 1 U of protease activity was defined as the amount of enzyme required to release 1 μg L-tyrosine per minute. L-tyrosine was used as standard for protease analysis. Enzyme activity was statistically analyzed via SPSS software. Each measurement was performed in triplicate. Furthermore, discs were obtained from 2-day-old cultures of *T*. *asperellum* GDFS1009 using sterile 3 mm hole-punchers. Each disc was inverted and placed on the center of a 9-cm Petri dish with 1.5% skim milk and 2.5% agar. After incubation at 28°C for 36h, proteolytic circle was observed [26–27].

Tobacco (*Nicotiana benthamiana*) plants and cucumber plants were grown to the 6–8-leaf stage in soil under a 10-h light/14-h dark cycle in a 24°C phytotron. A total of 20 μL of filtered xylanase preparation from *T*. *asperellum* GDFS1009 or blank broth (control) were injected into tobacco or cucumber leaves using 1 mL syringes. Injected leaves in each group were of the same size and position in the same tobacco or cucumber plant. The inoculated plant was placed in darkness in a 24°C phytotron, and tissue necrosis was observed every 12 h. For oxidative burst assay, injected leaves were stained in DAB solution (pH 3.8) for 8 h in darkness, boiled in ethanol, glycerin, and acetic acid (1:1:1, v/v) for 10–15 min, and washed twice with 60% ethanol [28].

### Global analysis of gene expression based on RNA-seq

The spore suspension of *T*. *asperellum* GDFS1009 was transferred into PD medium with a final spore concentration of 10^6^ cfu/mL. After culturing at 28°C and 180 rpm for 24 h, the mycelium was collected by vacuum filtration. Single-end (1×50) Solexa sequencing was performed, producing 20 million reads per sample. Additionally, 48-h samples were similarly sequenced, producing 10 million reads.

The reference genome for transcriptome analysis was acquired from http://genome.jgi.doe.gov/Trias1/Trias1.download.html. Conventional analysis was performed on the transcriptome data, including data pre-processing, genomic mapping, gene expression analysis, transcript expression analysis, alternative splicing analysis, analysis of differentially expressed genes, and GO/KEGG enrichment analysis of differentially expressed genes and non-differentially expressed genes [29–31]. On the basis of these results, genes related to mycoparasitism, induced resistance, and antibiosis in other *Trichoderma* strains were downloaded, and then local BLAST was used to compare them to the public *T*. *asperellum* genomic data mentioned above. Then all homologous genes were assembled and matched to the RNA-seq data of *T*. *asperellum* GDFS1009 to determine their expression levels. When the FPKM value exceeded 100, the expression level was defined as “high”, while an FPKM value of less than 10 was defined as “low”, and a value less than 1 was considered “ultra-low” expression. A heat map was constructed in the R language [32].

### Assay of primary metabolites based on GC-MS

The mycelia of *T*. *asperellum* GDFS1009 were inoculated into a PDA plate at 28°C for 4 d. Then, spores were collected in sterile water and diluted to 10^8^ cfu/mL. Spores (1 mL) were added to 100 mL PD broth and incubated on a 180-rpm rotary shaker at 28°C for 1 d. Next, 50 mL of this sample was dropped directly into 200 mL 60% (v/v) methanol solution (taken from a -40°C deep freezer). The mixture was allowed to cool for 10 min to allow the temperature to return to -40°C. The mixture was then centrifuged at 5000 rpm for 5 min at -10°C, and the mycelium pellet was collected. Following this procedure, the mycelium pellet was freeze-dried at -40°C.

Subsequently, 500 μL 100% methanol at -80°C was added to 100 mg of sample, vortexed for 30 s, and placed in liquid nitrogen for 15 min. After thawing at 4°C for 15 min and vortexing for 1 min, the mixture was centrifuged at 13000 rpm for 5 min at 4°C, and the resulting supernatant was transferred to tube A. Milli-Q water (250 mL) was used to resuspend the pellet after centrifugation. The resuspension was then vortexed for 30 s and placed in liquid nitrogen for 15 min. After thawing at 4°C for 15 min, glass beads (G8772-100G, Sigma, St. Louis, MO, USA) were added and vortexed for 10 min, and then the mixture was centrifuged at 13000 rpm for 5 min at 4°C. The supernatant was also transferred to tube A. After drying with nitrogen, 60 μL methoxyl amine hydrochloride (15 mg/mL) was added to tube A and vortexed for 1 min, and then the mixture was allowed to react at 25°C for 12 h. After adding 60 μL MSTFA with 1% TMCS and allowing the reaction occur at room temperature for 1 h, the mixture was centrifuged at 13000 rpm for 5 min at 4°C.

The supernatant was then used for gas chromatography-mass spectrometry (GC-MS; Agilent 7890A/5975C, Agilent, Santa Clara, CA, USA). The derivatized samples (1 μL) were injected at a 1:20 split ratio onto a HP-5MS column (5% phenyl methyl silox; 30 m × 250 μm i.d., 0.25-μm; Agilent J&W Scientific, Folsom, CA, USA). During GC, helium was supplied at a constant rate of 1 mL/min. The temperature of the injection port was 280°C, the ion source temperature was 250°C, and the interface temperature was 150°C. The temperature program started at 40°C for 5 min and increased at a rate of 10°C/min until it reached 300°C, where it was held constant for 5 min. MS was determined using the full-scan method with a range from 35 to 780 m/z. Identification of metabolites was performed in six biological replicates. Bioinformatics analyses included data pre-processing (XCMS, www.bioconductor.org) and compound identification (NIST 2008, Wiley 9) [33–35].

### Assay of secondary metabolites based on GC-MS

The mycelia of *T*. *asperellum* GDFS1009 were inoculated into PDA plate at 28°C for 6 d. After conidia fully covered the plate, they were carefully scraped from the plate surface with a sterile spoon. Spores (6 g) were weighed and extracted with 50 volumes of dichloromethane at 4°C for 2 d. The extract was exposed to 5% activated carbon under oscillation for 2 h and filtered with four layers of sterilized gauze. The supernatant was washed twice with an equal volume of 3% sodium carbonate solution. The resulting dichloromethane fraction was dehydrated with anhydrous sodium sulfate and then filtered with fast filter paper. Finally, the dehydrated liquid was vacuum-evaporated at 40°C to obtain 1 mL of viscous crude extract [36]. GC-MS analysis was carried out using an Auto System XL GC/Turbo Mass MSUSA (Perkin Elmer, USA, DB-5MS column: 30 m×0.25 mm, 0.25 μm). The GC conditions were as follows: after injection of 1 μL, the initial column temperature was held at 50°C for 5 min and then raised to 300°C at 5°C/min, and the vaporizing chamber temperature was then maintained at 300°C. The GC was operated with a split ratio of 10:1 using helium as the carrier gas. The electron-ionization (EI) source was held at 230°C and the ionization voltage was 70 eV. The quadrupole mass filter was run at an effective scanning range of 290–500 amu. Metabolites were qualitatively analyzed by GC-MS. The peak area of each metabolite, acquired in SIM mode (selective ion monitoring; signal/noise > 5.0), was normalized to the peak area of 2-heptone prior to further data processing.

Then spore suspension (10^6^ cfu/mL) of *T*. *asperellum* GDFS1009 was cultured in PD broth in a rotary shaker at 28°C and 180 rpm for 6 d. A total of 20 mL of filtered fermentation liquor from *T*. *asperellum* GDFS1009 (PD broth as control) was enriched to 5 mL, extracted three times with 5mL dichloromethane, and was washed twice with an equal volume of 3% sodium carbonate solution. The resulting dichloromethane fraction was vacuum-evaporated at 40°C to obtain 1 mL of viscous crude extract, and then was dehydrated with anhydrous sodium sulfate. Gas chromatography was performed on a HP-5MS capillary column (5% phenyl/95% methylpolysiloxane (30 m × 250 μm i.d., 0.25 μm film thickness, Agilent J & W Scientific, Folsom, CA, USA) to separate the derivatives at a constant flow of 1 mL/min helium. 1 μL of sample was injected in split mode in a 20:1 split ratio by the auto-sampler. Injection temperature was 280°C, the interface set to 150°C and the ion source adjusted to 230°C. The programs of temperature-rise was followed by initial temperature of 80°C for 5 min, 20°C/min rate up to 300°C and staying at 300°C for 6 min. Mass spectrometry was determined by full-scan method with range from 35 to 500 (m/z). Identification of metabolites using the Automatic Mass Spectral Deconvolution and Identification System (AMIDS) was searched against commercial available databases such as National Institute of Standards and Technology (NIST) and Wiley libraries [33–35].

### Assay of antimicrobial peptides based on UPLC-QTOF-MS/MS

Mycelia of *T*. *asperellum* GDFS1009 were inoculated onto a PDA plate at 28°C for 4 d. Then, spores were collected with sterile water to 10^8^ cfu/mL. Spores (1 mL) were added to 100 mL antimicrobial peptide media [0.5% glucose, 0.08% KH_2_PO_4_, 0.07% KNO_3_, 0.02% Ca(H_2_PO_4_)_2_, 0.05% MgSO_4_.7H2O, 0.001% MnSO_4_.5H_2_O, 0.0005% CuSO_4_.5H_2_O, and 0.0001% FeSO_4_.7H2O], and the mixture was incubated on a 180-rpm rotary shaker at 28°C for 20 d. The broth was filtered using two layers of qualitative filter paper, extracted with a filtrate:butanol ratio of 3:1, and the lower layer liquid was transferred to a vacuum distillation flask. After distillation, the dry powder was completely dissolved in 80 mL of methanol:dichloromethane at a ratio of 1:1, and then the dissolved liquid was filtered with a 0.45-μm PTFE membrane. After evaporating to dryness in a vacuum distillation flask, the solution was completely dissolved in 5 mL of dichloromethane:methanol at a ratio of 85:15 in a column that had been previously washed with 85:15 dichloromethane:methanol to remove acetone. This was followed by the addition of 100 mL of 85:15 dichloromethane:methanol. The collected filtrate was again placed in a vacuum distillation flask and evaporated to dryness. The dry powder was completely dissolved in 5 mL of methanol:H_2_O at a ratio of 85:15, transferred to a 1.5-mL centrifuge tube, and centrifuged at 13000 rpm for 10 min. After centrifugation, the supernatant was stored in a 2.0-mL glass vial at 4°C [37].

UPLC-QTOF-MS/MS was carried out using an ACQUITY UPLC & SCIEX SelexION Triple Quad^TM^ 5500 System (Waters, USA) equipped with an Acquity BEH C18 column (2.1 × 100 mm, 1.7 μm). The UPLC conditions were as follows: the mobile phase consisted of water (eluent A) and 0.1% acetonitrile (eluent B), the column temperature was 45°C, the injection volume was 3.00 μL, and the running time was 26.50 min. MS parameters were as follows: the spectrometer was operated in the positive ion mode using an electrospray-ionization source, the capillary voltage was 3.0 kV, the sampling cone voltage was 4.0 V, the extraction cone voltage was 3.0 V, the ion source temperature was 105°C, the atomization air temperature was 350°C, the atomizing gas flow was 700.0 L/h, the collision voltage was 3.0 eV, the scan time was 0.300 s, the scan range was 200–2200 m/z, and LockSpray online correction was performed using 200 ng/mL leucine enkephalin (556.2771 m/z). MassLynx software was used for the alignment, fitting, identification, and analysis of different antimicrobial peptides.

## Results

### Colony morphology, ITS identification, and phylogenetic tree

**Fig 1:**
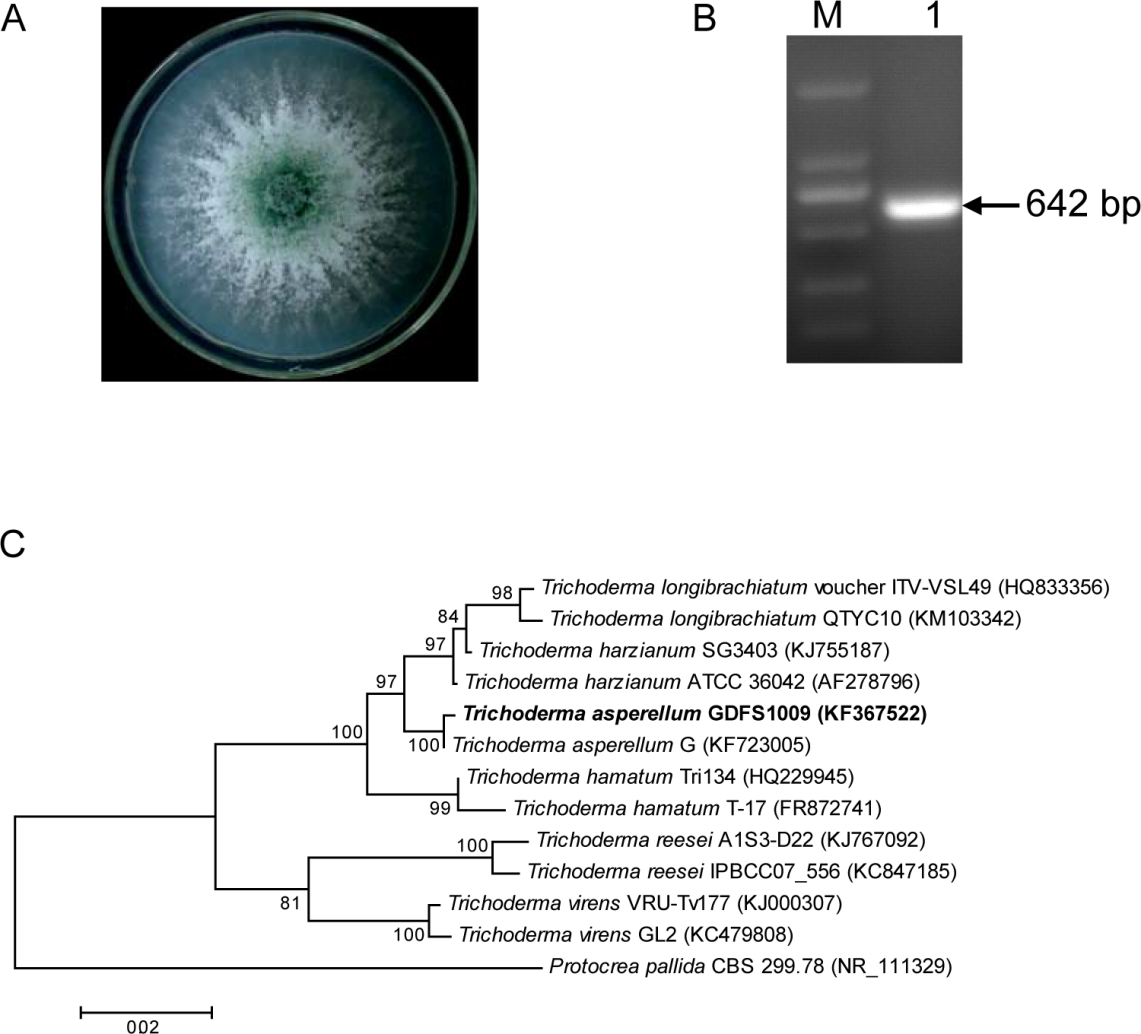
Morphological, ITS and phylogeny analysis of *T*. *asperellum* GDFS1009. (A) Photograph of colonial morphology on PDA medium; (B) ITS amplification; M, Marker DL2000 (Takara, Japan); 1, Band of ITS; (C) Phylogenetic tree based on nrDNA ITS sequence of *T*. *asperellum* GDFS1009 (bold) and its closely related species and outgroup retrieved from the literature.

Morphological observations showed that after five days of culture, the front of the novel isolate’s colony was dark green (spore color) and the back was white (mycelium color). The morphology of the mycelium was coarse, and dark green spores began to form in the center of the colony at high rates (Fig 1A). Based on preliminary morphological observations, the strain was confirmed to be *Trichoderma*.

The result of the PCR amplification of the ITS (KF367522) is showed in Fig 1B, with a product size of 642 bp. NCBI BLAST followed by phylogenetic analysis of ITS sequences revealed that this novel strain shares the highest homology with *T*. *asperellum* G, followed by *T*. *harzianum*, *T*. *longibrachiatum*, *T*. *hamatum*, *T*. *reesei*, and *T*. *virens*, with *Protocrea pallida* used as an outgroup (Fig 1C). Together with the morphological identification, we determined the strain to be *T*. *asperellum* and named it GDFS1009.

### Biocontrol effects under *in vitro* and greenhouse conditions

**Fig 2:**
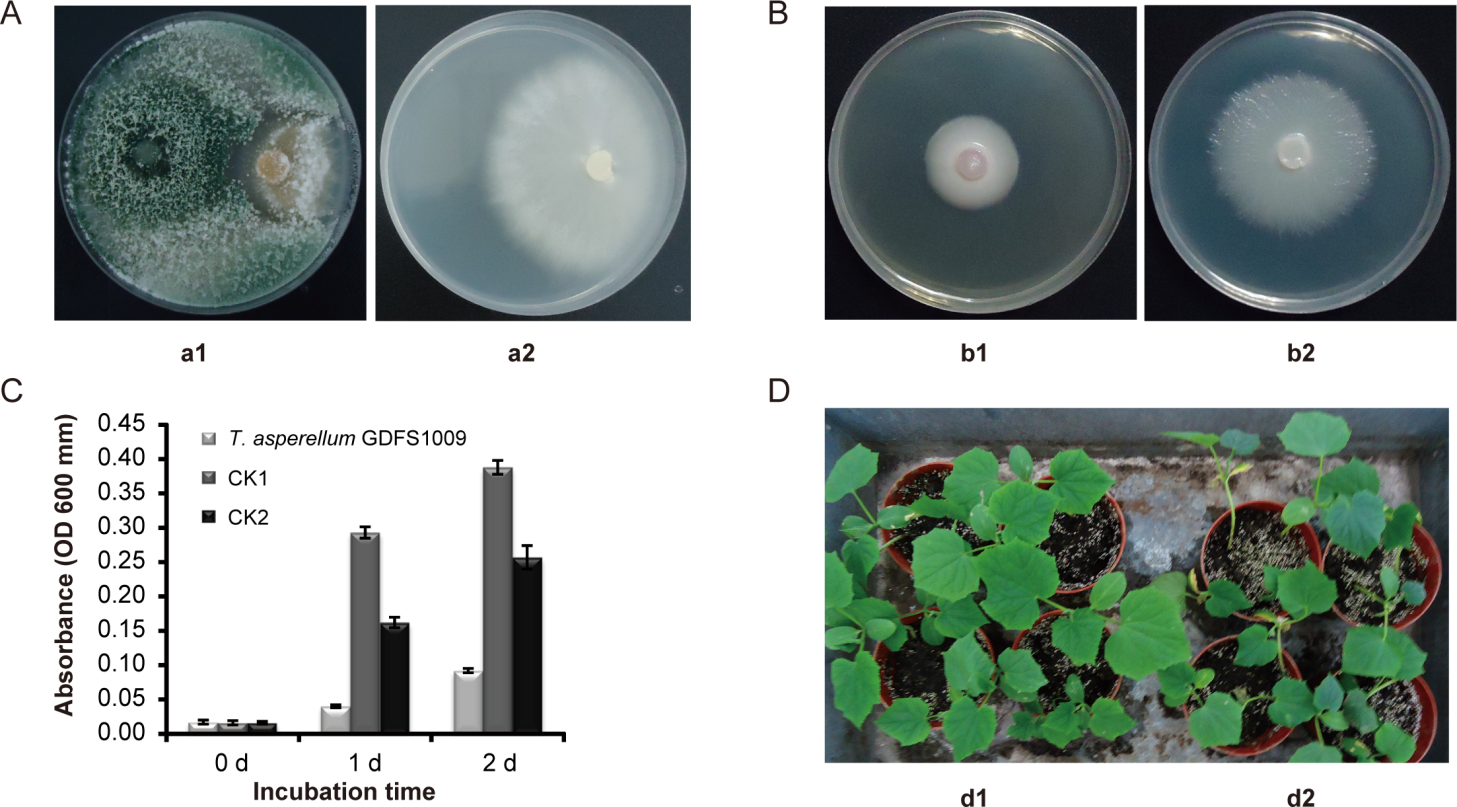
Control efficacy of *T*. *asperellum* GDFS1009 on *F*. *oxysporum* f. sp. *cucumerinum* Owen on cucumber. (A) Dual-culture assay, a1, Synergistic effect, a2, CK; (B) Resistant-dish assay, b1, Synergistic effect, b2, CK; (C) Micro well dilution assay; (D) Assay in green house, d1, Synergistic effect, d2, CK.

*In vitro* dual-culture analysis showed that the inhibition rate of *T*. *asperellum* GDFS1009 against *F*. *oxysporum* f. sp. *cucumerinum* Owen was 80.82% (Fig 2A). The inhibition rate of filtered fermentation liquor from *T*. *asperellum* GDFS1009 against *F*. *oxysporum* f. sp. *cucumerinum* Owen was 67.59% (Fig 2B). Micro well dilution assay on 2 days showed consistent results, up to 76.28% (Fig 2C). Under greenhouse conditions, *T*. *asperellum* GDFS1009 exhibited 86.34% growth inhibition (Fig 2D). Based on the *in vitro* antagonism assay and greenhouse study, *T*. *asperellum* GDFS1009 exhibits significant biocontrol effects on *F*. *oxysporum* f. sp. *cucumerinum* Owen. Additionally, except in the case of a low incidence of fusarium wilt, after treatment with *Trichoderma* spore suspension, cucumber seedling growth was more robust, with bright green leaves and strong roots (Fig 2D).

Comparing with *F*. *oxysporum* f. sp. *cucumerinum* Owen, *in vitro* dual-culture analysis showed that the inhibition rate of *T*. *asperellum* GDFS1009 against *F*. *graminearum* was not so high. However, the inhibition rate of filtered fermentation liquor from *T*. *asperellum* GDFS1009 against *F*. *graminearum* was 100%. Micro well dilution assay on 2 days showed consistent results, nearly 100% (S1 Fig).

### Mycoparasitism-related enzyme gene expression and activity

**Fig 3:**
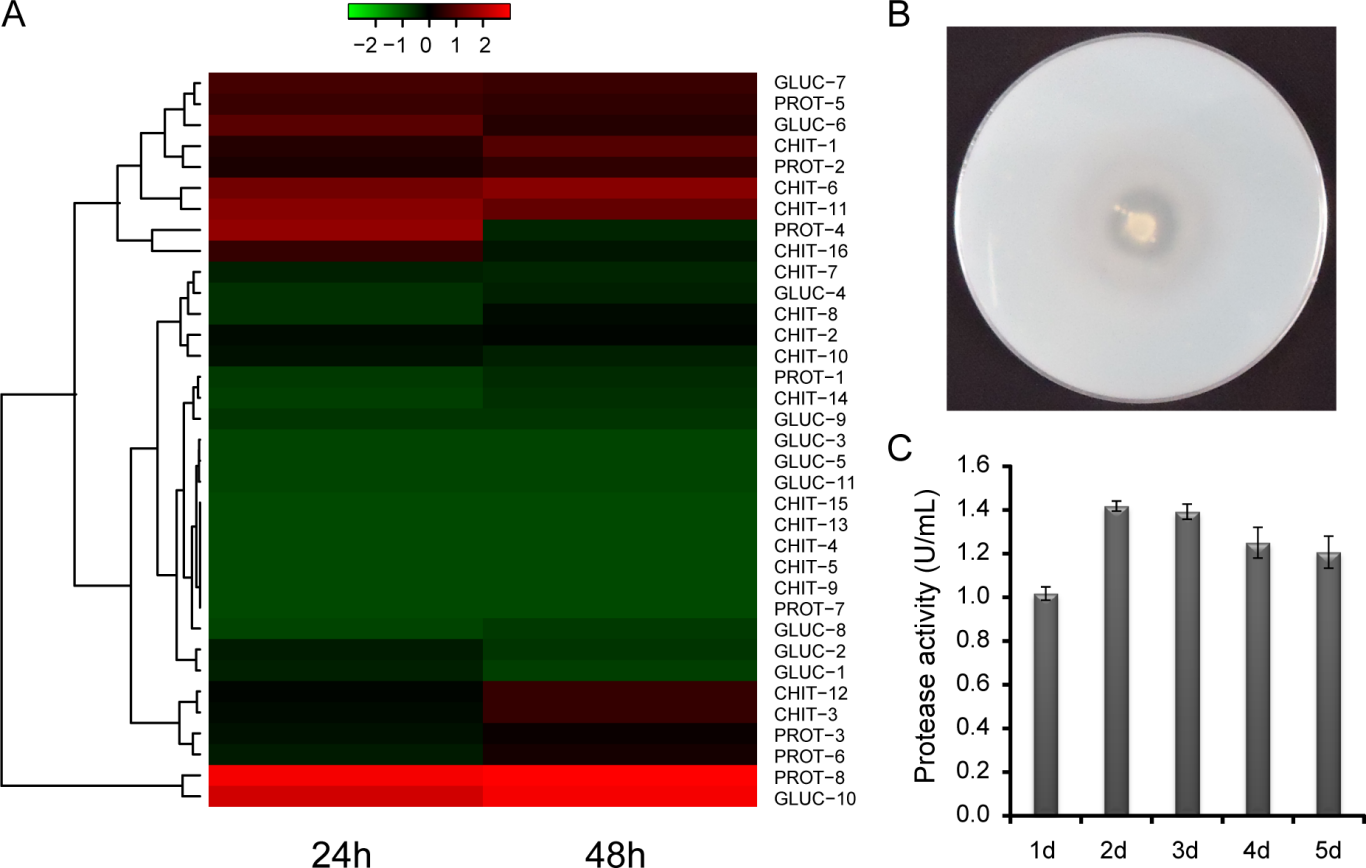
Protease analysis related to mycoparasitism in *T*. *asperellum* GDFS1009. (A) Heatmap of CWDEs expression based on RNA-seq; (B) Proteolytic circle assay; (C) Protease activity.

The relative expression levels of mycoparasitism genes were analyzed using RNA-seq analysis and illustrated with a heat map. Local BLAST identified 16 chitinase genes in the *T*. *asperellum* GDFS1009 genome and eight protease genes, with two being highly expressed after 24 h and one after 48 h. A total of 11 glucanase genes were discovered, with only one of these being highly expressed after 24 h and 48 h. Most of the non-highly expressed genes mentioned above were expressed at low levels, and a few did not appear to be expressed under the experimental conditions (Fig 3A and S1 Table).

After incubation at 28°C for 36h, proteolytic circle in skim milk plate was 1.656 ± 0.030 cm (Fig 3B). Activity of mycoparasitism-related protease was assessed in corresponding induction broth. And protease activity was highest after fermentation for 2 d, reaching 1.418 ± 0.023 U/mL at 50°C and pH 7.5 (Fig 3C). In addition, chitinase and glucanase were also discovered with high level expression in induction broth (Data not shown).

### Induced resistance-related gene expression and induction of tissue necrosis and resistance in plants

**Fig 4:**
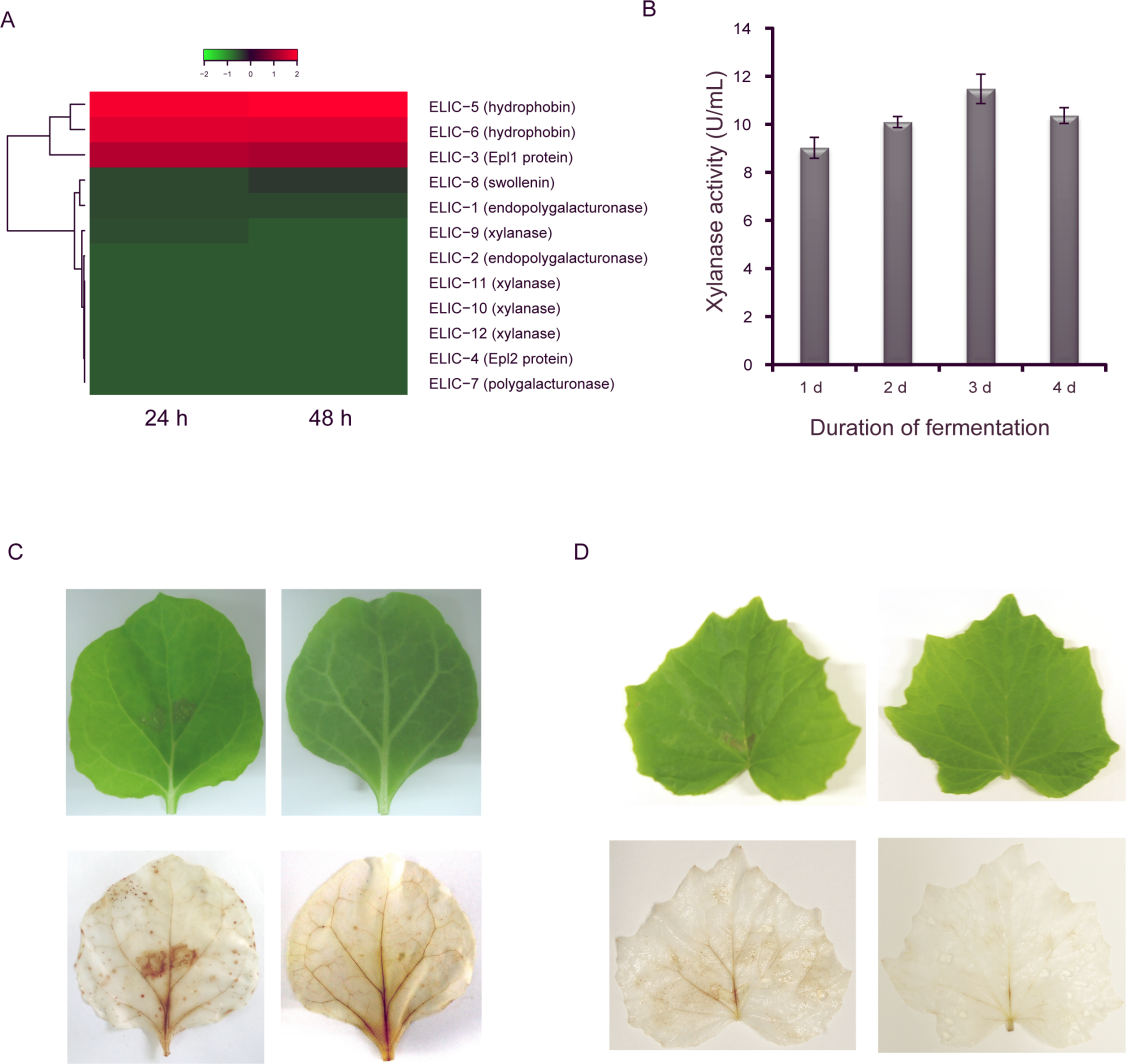
Induced resistance gene analysis of *T*. *asperellum* GDFS1009. (A) Heatmap of induced resistance gene expression based on RNA-seq; (B) Xylanase acvitity; (C) Photographs of tissue necrosis and DAB staining in tobacco leaves after inoculation for 24 h, c1 and c3, Tobacco leaf injected with xylanase preparation of *T*. *asperellum* GDFS1009, c2 and c4, Tobacco leaf injected with supernatant of blank broth (control); (D) Photographs of tissue necrosis and DAB staining in cucumber leaves after inoculation for 24 h, d1 and d3, Cucumber leaf injected with xylanase preparation of *T*. *asperellum* GDFS1009, d2 and d4, Cucumber leaf injected with supernatant of blank broth (control).

In the *T*. *asperellum* GDFS1009 genome, 12 elicitor genes with different functions were found, including two endopolygalacturonase genes, two Epl protein genes, two hydrophobin genes, one polygalacturonase gene, one swollenin gene, and four xylanase genes. Most of the elicitor genes were expressed at very low levels or not at all, with only three being expressed highly in PD broth after 24 h and 48 h (Fig 4A and S2 Table). The xylanase activity of *T*. *asperellum* GDFS1009 was assessed in induction broth, and exhibiting a maximum activity of 11.477 ± 0.613 U/mL at 50°C and pH 5.0 after fermentation for 3 d (Fig 4B).

At 12 h after inoculation, necrotic lesions were seen on tobacco and cucumber leaves injected with a xylanase solution from *T*. *asperellum* GDFS1009, but no necrotic lesions were seen on leaves treated with control broth. This result was most apparent at 24 h. In addition, sites both near and far from the inoculation sites of tobacco and cucumber leaves injected with xylanase solution were stained by DAB, while those in tobacco and cucumber leaves injected with control broth exhibited nearly no staining (Fig 4C and 4D).

### Biocontrol-related primary metabolites determined by GC-MS analysis

Using GC-MS analysis, nine were related to biocontrol as fungicides or a precursor/ intermediate of a fungicide, insecticide, or herbicide. Acetamide and diethylamine are fungicide precursors. Ethylene Glycol, ethanolamine, and o-toluic acid are fungicide intermediates. Citric acid is a fungicide. Ethylamine is a precursor of triazine herbicides, and glycine is a precursor of fungicides, insecticides, and herbicides (Table 1).

**Table 1:** Bio-control related primary metabolites in *T*. *asperellum* GDFS1009 based on GC-MS analysis.

Name	Formula	Function
Acetamide	C_2_H_5_NO	Fungicide precursor
Ethylamin	C_2_H_7_N	Precursor of triazine herbicides
Diethylamine	C_4_H_12_NO_4_P	Fungicide precursor
Ethylene Glycol	(CH_2_OH)_2_	Fungicide intermediate
Glycine	C_2_H_5_NO_2_	Precursor of fungicide, insecticide and herbicide
Ethanolamine	C_2_H_7_NO	Fungicide intermediate
O-Toluic acid	C_8_H_8_O_2_	Fungicide intermediate
Malic acid	C_4_H_6_O_5_	Precursor of insectifuge
Citric acid	C_6_H_8_O_7_	Fungicide

### Secondary metabolites determined by GC-MS analysis and gene cluster prediction

**Fig 5:**
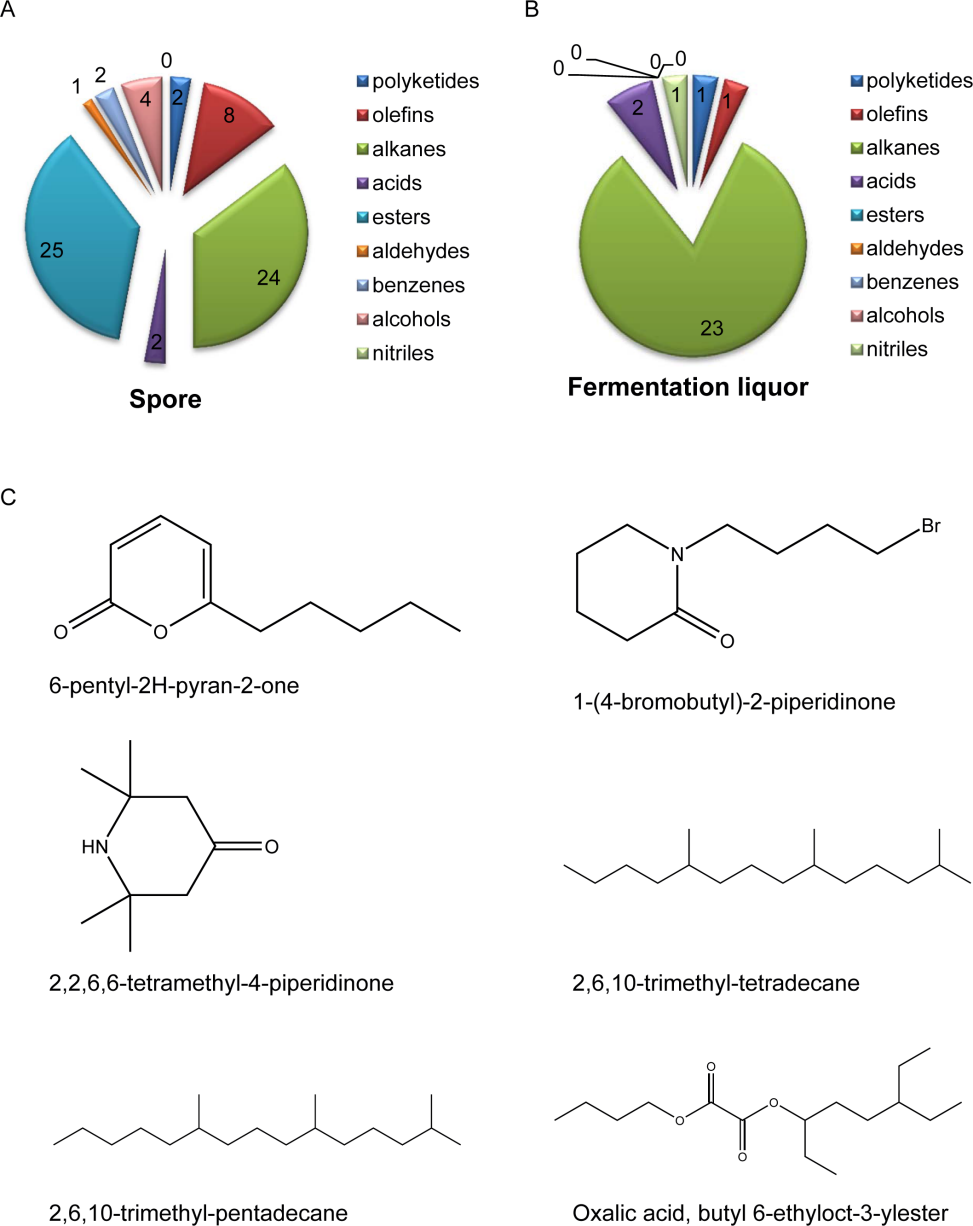
Antibiosis secondary metabolites in *T*. *asperellum* GDFS1009 based on GC-MS analysis. (A) Secondary metabolites in spore of *T*. *asperellum* GDFS1009; (B) Secondary metabolites in fermentation liquor of *T*. *asperellum* GDFS1009; (C) Some structures of polyketides, alkanes and acids related to pathogen resistance and induced resistance.

In spore of *T*. *asperellum* GDFS1009, 68 compounds were obtained via GC-MS analysis, including 2 polyketides, 8 olefins, 24 alkanes, 2 acids, 25 esters, 1 aldehydes, 2 benzenes, 4 alcohols (Fig 5A and S3 Table). In fermentation liquor of *T*. *asperellum* GDFS1009, 28 compounds were obtained via GC-MS analysis, including 1 polyketides, 1 olefins, 23 alkanes, 2 acids, 1 nitriles (Fig 5B and S4 Table).

1-(4-bromobutyl)-2-piperidinone, 2,2,6,6-tetramethyl-4-piperidinone, 6-pentyl-2H-pyran-2-one, 2,6,10-trimethyl-tetradecane, 2,6,10-trimethyl-pentadecane, and oxalic acid, butyl 6-ethyloct-3-ylester in *T*. *asperellum* GDFS1009 were found, which may be related with pathogen inhibition and induced resistance (Fig 5C).

Local BLAST revealed 16 gene clusters of polyketide synthases (PKSs) in the *T*. *asperellum* GDFS1009 genome, among which 12 clusters were fully reducing PKSs and four were non-reducing PKSs. Based on the analysis of RNA-seq data, after 24 h and 48 h in PD broth, almost all of the gene clusters exhibited low levels of expression or were not expressed (Table 2).

**Table 2:** Gene cluster analysis of polyketide and nonribosomal peptides synthesis in *T*. *asperellum* GDFS1009.

Scaffold	Name	Homolog accession number	Species with the homolog	Homology	FPKM	FPKM
					-24h	-48h
scaffold_1–1	FR-PKS-1	KLP09988.1	*Fusarium fujikuroi*	59%	0.860	0.880
scaffold_13–1	FR-PKS-2	XP_013945032.1	*Trichoderma atroviride*	97%	0.040	0.000
scaffold_15–1	FR-PKS-3	XP_013945595.1	*Trichoderma atroviride*	82%	2.110	1.090
scaffold_15–2	FR-PKS-4	XP_013945816.1	*Trichoderma atroviride*	83%	0.010	0.090
scaffold_16–2	FR-PKS-5	XP_013940837.1	*Trichoderma atroviride*	89%	0.090	0.080
scaffold_18	FR-PKS-6	XP_006966953.1	*Trichoderma reesei*	81%	0.140	0.050
scaffold_2	FR-PKS-7	KUE98568.1	*Trichoderma gamsii*	88%	4.400	37.720
scaffold_21	FR-PKS-8	XP_013949124.1	*Trichoderma atroviride*	91%	0.370	0.650
scaffold_5–2	FR-PKS-9	OAQ76947.1	*Purpureocillium lilacinum*	47%	0.870	1.020
scaffold_5–3	FR-PKS-10	XP_013938986.1	*Trichoderma atroviride*	97%	0.070	0.050
scaffold_5–5	FR-PKS-11	XP_013940269.1	*Trichoderma atroviride*	89%	14.830	20.690
scaffold_16–3	FR-PKS-12	GAT21130.1	*Aspergillus luchuensis*	63%	0.600	1.500
scaffold_26	NR-PKS-1	KJK76275.1	*Metarhizium anisopliae*	75%	0.710	1.910
scaffold_27	NR-PKS-2	KNG44542.1	*Stemphylium lycopersici*	64%	0.010	0.000
scaffold_5–4	NR-PKS-3	KUF03965.1	*Trichoderma gamsii*	85%	0.890	3.280
scaffold_6–3	NR-PKS-4	XP_013944236.1	*Trichoderma atroviride*	83%	0.300	0.100
scaffold_1–3	NRPS-1	XP_013939839.1	*Trichoderma atroviride*	97%	3.260	5.300
scaffold_10	NRPS-2	XP_013942944.1	*Trichoderma atroviride*	81%	15.310	7.600
scaffold_11	NRPS-3	KLP12855.1	*Fusarium fujikuroi*	52%	0.450	0.290
scaffold_1–2	NRPS-4	XP_013948911.1	*Trichoderma atroviride*	72%	0.110	0.180
scaffold_13–2	NRPS-5	XP_007813200.1	*Metarhizium acridum*	72%	0.000	0.000
scaffold_1–4	NRPS-6	XP_013938832.1	*Trichoderma atroviride*	82%	1.580	0.010
scaffold_28	NRPS-7	XP_013949249.1	*Trichoderma atroviride*	85%	7.060	0.830
scaffold_29	NRPS-8	KOC17602.1	*Aspergillus flavus*	56%	0.180	0.110
scaffold_5–1	NRPS-9	XP_009157203.1	*Exophiala dermatitidis*	36%	0.060	0.010
scaffold_5–6	NRPS-10	XP_006964610.1	*Trichoderma reesei*	65%	0.040	0.010
scaffold_5–7	NRPS-11	GAO86329.1	*Aspergillus udagawae*	57%	3.200	0.820
scaffold_6–1	NRPS-12	XP_013954993.1	*Trichoderma virens*	66%	0.090	0.330
scaffold_6–2	NRPS-13	XP_013944039.1	*Trichoderma atroviride*	79%	0.230	1.180
scaffold_6–4	NRPS-14	XP_013952882.1	*Trichoderma virens*	82%	1.900	1.140
scaffold_8	NRPS-15	XP_013960079.1	*Trichoderma virens*	42%	0.100	0.060
scaffold_30	NRPS-16	OBT62387.1	*Pseudogymnoascus* sp.	60%	0.410	0.840
scaffold_7	NRPS-PKS-1	XP_013957420.1	*Trichoderma virens*	55%	0.240	1.880
scaffold_16–1	NRPS-PKS-2	XP_013330927.1	*Rasamsonia emersonii*	73%	15.120	12.070

### Antimicrobial peptides determined by UPLC-QTOF-MS/MS analysis and gene cluster prediction

Through UPLC-QTOF-MS/MS analysis, a total of six types of antimicrobial peptides were speculated from *T*. *asperellum* GDFS1009 with molecular weights ranging from 1092.6450 to 1734.0618. These were TBV (11 aa), trichotoxin_A-50_F (18 aa), hypomurocin_B_IIIa (18 aa), trichotoxin_A-50_I (18 aa), hypomurocin_B_I (18 aa), and hypomurocin_B_II (18 aa; Table 3). These antimicrobial peptides are formed through the catalysis of non-ribosomal peptide synthases (NRPS). Local BLAST revealed 16 NRPS gene clusters in the *T*. *asperellum* GDFS1009 genome, with two PKS/NRPS hetero-gene clusters. Based on the analysis of RNA-seq data, after 24 h and 48 h in PD broth, almost all of the gene clusters were lowly expressed or not expressed (Table 2).

**Table 3:** Peptaibols antimicrobial peptides in *T*. *asperellum* GDFS1009 based on UPLC-QTOF-MS/MS analysis.

Peptaibols	Molecular Formula	Molecular weight
TBV	Ac Aib Ser Vxx Vxx Aib Pro Lxx Lxx Aib Pro Aib OH	1092.6450
Trichotoxin_A-50_F	Ac Aib Gly Aib Lxx Aib Gln Aib Aib Ala Ala Ala Aib Pro Lxx Aib Vxx Gln Vxx OH	1690.0410
Hypomurocin_B_IIIa	Ac Aib Ala Ala Lxx Aib Gln Aib Vxx Aib Gly Aib Aib Pro Lxx Aib Aib Gln Vxx OH	1704.0449
Trichotoxin_A-50_I	Ac Aib Gly Aib Lxx Aib Gln Aib Aib Aib Ala Aib Aib Pro Lxx Aib Vxx Gln Vxx OH	1718.0642
Hypomurocin_B_I	Ac Aib Ser Ala Lxx Aib Gln Aib Vxx Aib Gly Aib Aib Pro Lxx Aib Aib Gln Vxx OH	1720.0493
Hypomurocin_B_II	Ac Aib Ser Ala Lxx Aib Gln Aib Vxx Aib Gly Aib Aib Pro Lxx Aib Aib Gln Lxx OH	1734.0618

## Discussion

In recent years, the United States, Canada, Australia, Poland, and many other countries have participated in collecting *Trichoderma* resources. At present, 141 *Trichoderma* species have been discovered in the world [38–41]. Early studies on *Trichoderma* mostly focused on aspects of soil microbiology and general mycology. As our understanding of *Trichoderma* has deepened, research into the mechanism of biocontrol and application has gradually increased.

This study began with the identification and biocontrol assessment of a novel *Trichoderma*, named *T*. *asperellum* GDFS1009, which exhibits strong control effects on *F*. *graminearum* and *F*. *oxysporum* (Figs 1 and 2; S1 Fig). As is typical of soil-borne diseases, *F*. *graminearum* and *F*. *oxysporum* seriously harm vegetables and food crops. The biocontrol data from *in vitro* and greenhouse experiments provide an experimental basis for further investigations into spore granule production for use in agricultural settings.

Previously, research into the resistance mechanisms of *Trichoderma* spp. have been fairly broad, with most concentrated on a single or a few biocontrol factors in a single strain. Our study reveals the global resistance mechanisms of *T*. *asperellum* GDFS1009 at the molecular level, including almost all biocontrol factors related to mycoparasitism, induced resistance, and antibiosis using genomics, transcriptomics, and metabolomics analyses as well as a series of physiological and biochemical analyses.

Chitinases, involved in mycoparasitism, are diverse, inducible secretory proteins. Characteristics of chitinases differ in different *Trichoderma* species. *Trichoderma koningii* inhibits the growth of *Sclerotium cepivorum* in onion root via the production of exo- and endo-chitinases, which digest the cell walls of pathogens [42]. β-1, 3-glucanase, also associated with mycoparasitism, induces strong hydrolysis of pathogen cell walls, thereby inhibiting germination of pathogen spores and digestion of the mycelium [43]. In addition, proteases play an important role in mycoparasitism, as they are necessary for the complete hydrolysis of pathogen cell walls [44]. In our study, 16 chitinase genes, eight protease genes, and 11 glucanase genes were discovered in *T*. *asperellum* GDFS1009, only a few of which were highly expressed in PD broth without induction medium (Fig 3 and S1 Table). In future experiments, the specific functions of these enzymes, such their tolerances to heat, cold, or metal ions, could be predicted with bioinformatics analysis or via expression in *E*. *coli* or *Saccharomyces cerevisiae* in order to more fully understand their enzymatic properties. In the induction broths, the three types of enzymes were all detected and exhibited high activities, providing an experimental basis for subsequent development of enzyme preparations. Induction broths should be optimized for greater enzyme activity, and single or mixed enzyme preparations may be applied in agricultural production.

In recent years, it was discovered that *Trichoderma* and its formulations could elicit pathogen-related gene expression in host plants and induce systematic acquired resistance (SAR). In 1999, Yedidia found that when the *Trichoderma* mycelium crosses the epidermis layer of cucumber root, it induces a cascade of pathogen-related protein expression, similar to that caused by the chemical mutagen 2, 6-dichlorphenoxyacetic acid [45, 46]. Similarly, after treatment of the cucumber root with *T*. *harzianum* T-203, the JA/SA signal pathway was induced, and *Pseudomonas lachrymans* was inhibited [47, 48]. Numerous studies have shown that *Trichoderma* produces several types of inducible factors or elicitors, such as *sm1* and xylanase. In this study, xylanase activity was also detected in induction broth. This may induce plant resistance due to the induction of tissue necrosis. Additionally, in the *T*. *asperellum* GDFS1009 genome, 12 elicitor genes with different functions were found, including two endopolygalacturonase genes, two Epl protein genes, two hydrophobin genes, one polygalacturonase gene, one swollenin gene, and four xylanase genes. The expression levels of most of these elicitor genes were very low or not detected, with only a few being highly expressed in PD broth after 24 h and 48 h (Fig 4 and S2 Table). In future experiments, elicitors which mechanisms are unknown could be expressed in *E*. *coli* or *Saccharomyces* and be purified for further investigation into the interactions with plants and pathogens.

Nine primary metabolites believed to be fungicides or precursors/intermediates of fungicides, insecticides, or herbicides were identified in *T*. *asperellum* GDFS1009 (Table 1). Acetamide is widely used as a pesticide. Dibromocyanoacetamide (DBNPA) is a novel potent sanitizer and excellent water treatment agent [49]. Ethylamine can be used as a raw material to produce triazine herbicides [50], while ethylene glycol can be used as an intermediate for pesticide production [51]. Glycine can be used to synthesize fungicides, and research has also shown that copper glycine inhibits *B*. *cinerea* and *F*. *oxysporum* [52]. Ethanolamine is an intermediate in the synthesis of pesticides. The combined application of Schiff bases of salicylaldehyde ethanolamine with copper acetate or zinc sulfate exhibited significant inhibition of *Aspergillus niger* and *F*. *oxysporum* [53]. As an important organic synthesis intermediate, o-toluic acid is also widely used in the production of pesticides [54]. L-malic acid can be used for pharmaceutical formulations and also for the synthesis of insect repellents [55]. Citric acid strongly inhibits *Aeromonas hydrophila*, *Pseudomonas fluorescens*, and other aquatic pathogens [56].

*Trichoderma* also produces a variety of antimicrobial secondary metabolites, including diketopiperazines, sesquiterpenes, polyketides, olefins, alkanes and so on [57–58]. The biological activities of several antimicrobial substances have been identified [59–62]. In 1987, Claydon purified alkyl pyrone from *T*. *harzianum* [63]. Horace reported that a main mechanism of the inhibition of *Rhizoctonia solani* by *T*. *harzianum* involves the production of 6-pentyl pyrone [17]. It was found that 2,2,6,6-tetramethylpiperidine also has antifungal activity [64]. Furthermore, oxalic acid-induced resistance to *Rhizoctonia solani* in rice is associated with induction of phenolics, peroxidase and pathogenesis-related proteins [65]. By comparing the secondary metabolites in *T*. *asperellum* GDFS1009 to standards and databases, in spore of *T*. *asperellum* GDFS1009, 68 compounds were obtained via GC-MS analysis, including 2 polyketides, 8 olefins, 24 alkanes, 2 acids, 25 esters, 1 aldehydes, 2 benzenes, 4 alcohols (Fig 5A and S3 Table). In fermentation liquor of *T*. *asperellum* GDFS1009, 28 compounds were obtained via GC-MS analysis, including 1 polyketides, 1 olefins, 23 alkanes, 2 acids, 1 nitriles (Fig 5B and S4 Table). Comparative analysis of spore and fermentation liquor showed that: (1) there is no great difference about numbers of polyketides, alkanes and acids; (2) the number of olefins in spore is more than in fermentation liquor; (3) esters, aldehydes, benzenes and alcohols are unique to spore, and nitriles is unique to fermentation liquor. 1-(4-bromobutyl)-2-piperidinone, 2,2,6,6-tetramethyl-4-piperidinone, 6-pentyl-2H-pyran-2-one, 2,6,10-trimethyl-tetradecane, 2,6,10-trimethyl-pentadecane, and oxalic acid, butyl 6-ethyloct-3-ylester in *T*. *asperellum* GDFS1009 were found, which are related with pathogen inhibition and induced resistance (Fig 5C). Additionally, via bioinformatics analysis, 16 gene clusters of polyketide synthases were discovered in *T*. *asperellum* GDFS1009, almost all of which are lowly expressed or non-detectable after 24 h and 48 h in PD broth (Table 2).

Antimicrobial peptides exhibit broad antimicrobial abilities and strong non-specific resistance to pathogens. In 2005, Nielsen isolated an antimicrobial peptide polymer with 20 residues from *T*. *brevicompactum* [18]. In 2006, Kada isolated a new, short antimicrobial peptide from *Trichoderma* [66]. In 2007, Ruiz identified 11 residual fragments from antimicrobial peptides in *T*. *longibrachiatum* [19]. In this study, six types of antimicrobial peptides were detected and speculated from *T*. *asperellum* GDFS1009: TBV, trichotoxin_A-50_F, hypomurocin_B_IIIa, trichotoxin_A-50_I, hypomurocin_B_I, and hypomurocin_B_II (Table 3). As antimicrobial peptides are formed through the catalysis of NRPS, related gene clusters were analyzed and 16 NRPSs were found in *T*. *asperellum* GDFS1009, with two PKS/NRPS hetero-gene clusters. After 24 h and 48 h in PD broth, almost all of these gene clusters were lowly expressed or not detected (Table 2).

Based on these results, we infer that there are many mycoparasitism-related enzymes, elicitors, primary and secondary metabolites in *Trichoderma* that have not yet been discovered, including chitinase, protease, polyketides, antimicrobial peptides and so on. Future experiments should focus on optimizing the ingredients of the induction broth and the incubation conditions during the fermentation process. In addition, the silent genes and gene clusters should be expressed in *Saccharomycetes*. In conclusion, *Trichoderma* spp. offer great promise for ongoing agricultural development, expressing many promising enzymes, elicitors, and secondary metabolites.

## Conclusion

Due to its efficient broad-spectrum antimicrobial activity, *Trichoderma* has been established as an internationally recognized biocontrol fungus. Though researches about *Trichoderma* spp. were very broad, most just concentrated in one or several biocontrol factors in one strain. Our study reveals resistant molecular mechanisms of *T*. *asperellum* in global aspects, including almost all biocontrol factors related to mycoparasitism, induced resistance and antibiosis, based on genomics, transcriptomics, metabolomics and a series of physiological and biochemical analyses. Firstly, we found and identified a novel strain of *T*. *asperellum*, named GDFS1009. The mycelium of *T*. *asperellum* GDFS1009 has high growth rate, high sporulation capacity, and very strong inhibitory effects on pathogens that cause cucumber fusarium wilt and corn stalk rot. *T*. *asperellum* GDFS1009 secretes chitinase, glucanase and protease, which can degrade the cell walls of fungi and participate in the mycoparasitism. The secreted xylanase are good candidates in inducing plant resistance and enhancing plant immunity. Then, RNA-seq and GC-MS analysis showed that *T*. *asperellum* GDFS1009 can produce nine primary metabolites which are precursors of antimicrobial compounds, and a variety of antimicrobial secondary metabolites, including polyketides, alkanes and so on. In addition, this study speculated six antimicrobial peptides via UPLC-QTOF-MS/MS. However, through synthetic analysis we can infer that there are some enzymes and elicitors with specific function and lots of secondary metabolites in *Trichoderma* have not been discovered. Subsequently, these related genes or gene clusters could be predicted and expressed via *E*. *coli* and *Saccharomyces* systems for wider application in the field of agricultural biocontrol.

## Supporting information

S1 Fig Control efficacy of *T*. *asperellum* GDFS1009 on *F*. *gramimearum*. (A) Dual-culture assay, a1, Synergistic effect, a2, CK; (B) Resistant-dish assay, b1, Synergistic effect, b2, CK; (C) Micro well dilution assay.
(DOCX)

S1 Table Amino acid sequence homologies between mycoparasitism related enzymes in *T*. *asperellum* GDFS1009 and other strains. (DOCX)

S2 Table Amino acid sequence homologies between induced resistant related enzymes in *T*. *asperellum* GDFS1009 and other strains. (DOCX)

S3 Table Antibiosis secondary metabolites analysis in spore of *T*. *asperellum* GDFS1009. (DOCX)

S4 Table Antibiosis secondary metabolites analysis in fermentation liquor of *T*. *asperellum* GDFS1009. (DOCX)
