# Supplementary material for: Identification of a novel fungus, Trichoderma asperellum GDFS1009, and comprehensive evaluation of its biocontrol efficacy
Source: PLoS One. 2017 Jun 23;12(6):e0179957. doi: 10.1371/journal.pone.0179957 (PMC5482467; doi:10.1371/journal.pone.0179957)

**S1 Fig. Control efficacy of *T. asperellum* GDFS1009 on *F.* *gramimearum*.** (A) Dual-culture assay, a1, Synergistic effect, a2, CK; (B) Resistant-dish assay, b1, Synergistic effect, b2, CK; (C) Micro well dilution assay.


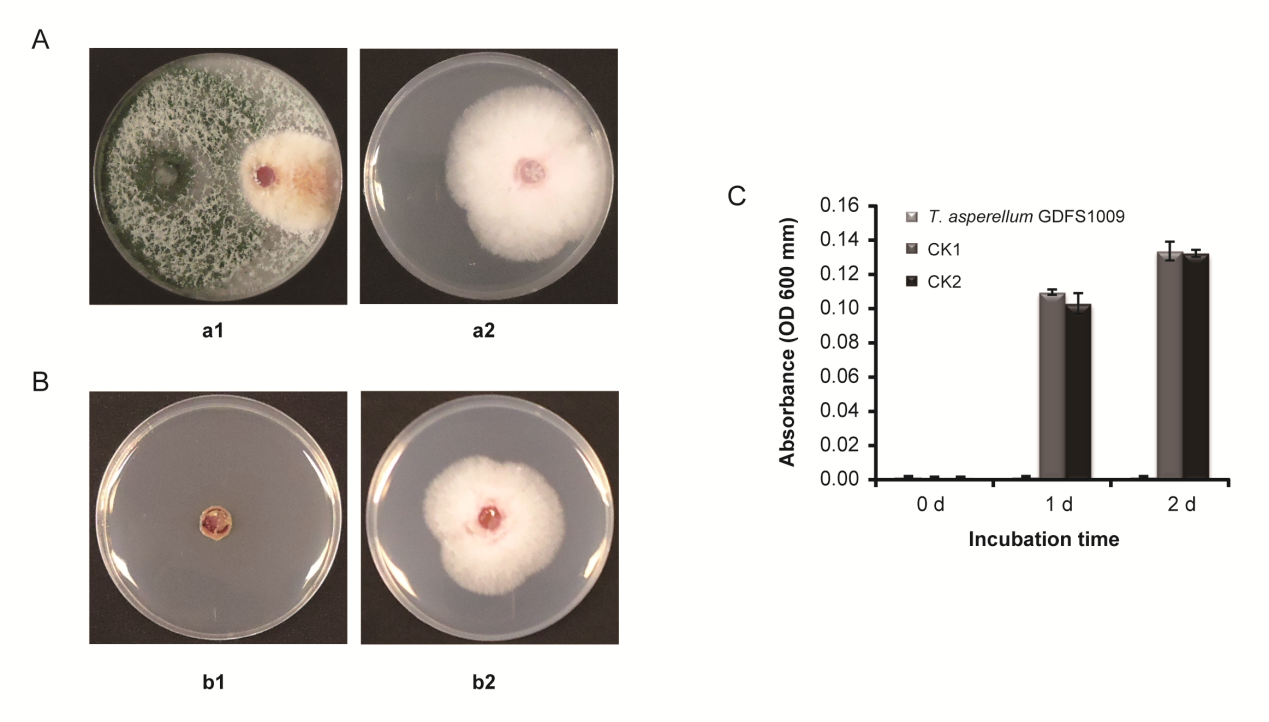

Supplement: S1 Fig — (A) Dual-culture assay, a1, Synergistic effect, a2, CK; (B) Resistant-dish assay, b1, Synergistic effect, b2, CK; (C) Micro well dilution assay. (DOCX) [file pone.0179957.s001.docx]
