# Supplementary material for: Identification of a novel fungus, Trichoderma asperellum GDFS1009, and comprehensive evaluation of its biocontrol efficacy
Source: PLoS One. 2017 Jun 23;12(6):e0179957. doi: 10.1371/journal.pone.0179957 (PMC5482467; doi:10.1371/journal.pone.0179957)
Supplement: S1 Table — (DOCX) [file pone.0179957.s002.docx]

**S1 Table. Amino acid sequence homologies between mycoparasitism related enzymes in *T. asperellum* GDFS1009 and other strains**

| **Location** | **Name** | **Function** | **Homolog accession number** | **Species with the homolog** | **Homology** | **FPKM-24h** | **FPKM- 48h** |
| --- | --- | --- | --- | --- | --- | --- | --- |
| scaffold 10 | PROT-1 | alkaline proteinase | KUF00488.1 | *Trichoderma gamsii* | 71% | 0.33 | 0.97 |
| scaffold 13-3 | PROT-2 | acid protease | ETR99661.1 | *Trichoderma reesei* | 80% | 9.37 | 13.79 |
| scaffold 2 | PROT-3 | acid protease | KUJ11569.1 | *Phialocephala scopiformis* | 74% | 2.36 | 5.66 |
| scaffold 5-3 | PROT-4 | alkaline proteinase | AAP15044.1 | *Trichoderma hamatum* | 90% | 148.34 | 1.30 |
| scaffold 7-2 | PROT-5 | protease | XP_006966748.1 | *Trichoderma reesei* | 81% | 20.11 | 13.00 |
| scaffold 1-2 | PROT-6 | proteinase | KJK74756.1 | *Metarhizium anisopliae* | 61% | 1.64 | 7.30 |
| scaffold 18-1 | PROT-7 | subtilisin-like protease | ABI84117.1 | *Trichoderma harzianum* | 89% | 0.00 | 0.00 |
| scaffold 3-3 | PROT-8 | vacuolar protease A | ACF20292 | *Trichoderma aureoviride* | 100% | 1225.71 | 1254.60 |
| scaffold 14 | CHIT-1 | chitinase 42 | ADI46579.1 | *Trichoderma asperellum* | 99% | 11.31 | 30.65 |
| scaffold 5-5 | CHIT-2 | chitinase | KUF01433.1 | *Trichoderma gamsii* | 96% | 2.76 | 3.14 |
| scaffold 8-3 | CHIT-3 | chitinase | ACI96032.1 | *Trichoderma virens* | 95% | 2.81 | 15.39 |
| scaffold 3-1 | CHIT-4 | chitinase | KUF00833.1 | *Trichoderma gamsii* | 94% | 0.00 | 0.00 |
| scaffold 12 | CHIT-5 | chitinase 1 | KIL83821.1 | *Fusarium avenaceum* | 78% | 0.00 | 0.00 |
| scaffold 11-1 | CHIT-6 | chitinase 18-2 | AAZ23946.1 | *Trichoderma atroviride* | 93% | 72.66 | 93.10 |
| scaffold 4-3 | CHIT-7 | chitinase 33 | ACJ04784.1 | *Trichoderma virens* | 90% | 1.33 | 1.28 |
| scaffold 13-1 | CHIT-8 | chitinase chi18-13 | ADF57303.1 | *Trichoderma tomentosum* | 88% | 0.83 | 2.71 |
| scaffold 13-5 | CHIT-9 | chitinase chi18-17 | ADF57312.1 | *Trichoderma lixii* | 73% | 0.00 | 0.00 |
| scaffold 5-1 | CHIT-10 | chitinase 36 | AGG86665.1 | *Trichoderma koningii* | 99% | 2.44 | 1.38 |
| scaffold 13-6 | CHIT-11 | class III chitinase | KUE97974.1 | *Trichoderma gamsii* | 90% | 115.44 | 44.08 |
| scaffold 1-1 | CHIT-12 | class V chitinase | AAL78812.1 | *Trichoderma virens* | 58% | 3.39 | 15.40 |
| scaffold 3-2 | CHIT-13 | endochitinase | ADZ48671 | *Trichoderma asperellum* | 98% | 0.00 | 0.00 |
| scaffold 4-2 | CHIT-14 | endochitinase 2 | KND88383.1 | *Tolypocladium ophioglossoides* | 58% | 0.26 | 0.84 |
| scaffold 8-1 | CHIT-15 | endochitinase 2 | KND91981.1 | *Tolypocladium ophioglossoides* | 64% | 0.00 | 0.00 |
| scaffold 8-2 | CHIT-16 | endochitinase 2 | KND88383.1 | *Tolypocladium ophioglossoides* | 60% | 15.91 | 1.97 |
| scaffold 19-1 | GLUC-1 | endo-1,3-β-glucosidase | KUF02974.1 | *Trichoderma gamsii* | 94% | 1.42 | 0.31 |
| scaffold 4-1 | GLUC-2 | endo-1,3-β-glucosidase | KUE98268.1 | *Trichoderma gamsii* | 94% | 1.68 | 0.51 |
| scaffold 7-4 | GLUC-3 | endo-1,3-β-glucosidase | KUF01083.1 | *Trichoderma gamsii* | 95% | 0.14 | 0.10 |
| scaffold 18-2 | GLUC-4 | endo-1,6-β-glucanase | ACE81431.1 | *Trichoderma lixii* | 87% | 0.78 | 1.55 |
| scaffold 19-2 | GLUC-5 | endo-1,6-β-glucosidase | KPM38233.1 | *Neonectria ditissima* | 61% | 0.10 | 0.09 |
| scaffold 16-4 | GLUC-6 | exo-1,3-β- glucanase | EQB47470.1 | *Colletotrichum gloeosporioides* | 50% | 40.30 | 9.93 |
| scaffold 1-3 | GLUC-7 | exo-1,3-β-glucanase | OAQ61185.1 | *Pochonia chlamydosporia* | 67% | 23.84 | 16.04 |
| scaffold 5-4 | GLUC-8 | exo-1,3-β-glucanase | OAQ59871.1 | *Pochonia chlamydosporia* | 70% | 0.07 | 0.43 |
| scaffold 9-2 | GLUC-9 | exo-1,3-β-glucanase | OAQ67089.1 | *Pochonia chlamydosporia* | 72% | 0.60 | 0.56 |
| scaffold 1-4 | GLUC-10 | exo-1,3-β-glucanase | KLP03973.1 | *Fusarium fujikuroi* | 34% | 573.68 | 965.91 |
| scaffold 16-3 | GLUC-11 | exo-1,3-β-glucanase | ABY19519.1 | *Trichoderma asperellum* | 94% | 0.10 | 0.14 |
