# Supplementary material for: Identification of a novel fungus, Trichoderma asperellum GDFS1009, and comprehensive evaluation of its biocontrol efficacy
Source: PLoS One. 2017 Jun 23;12(6):e0179957. doi: 10.1371/journal.pone.0179957 (PMC5482467; doi:10.1371/journal.pone.0179957)
Supplement: S2 Table — (DOCX) [file pone.0179957.s003.docx]

**S2 Table. Amino acid sequence homologies between induced resistant related enzymes**

**in *T. asperellum* GDFS1009 and other strains**

| **Location** | **Name** | **Function** | **Homolog accession number** | **Species with the homolog** | **Homology** | **FPKM-24h** | **FPKM-48h** |
| --- | --- | --- | --- | --- | --- | --- | --- |
| scaffold 16-2 | ELIC-1 | endopolygalacturonase | AHK61121.1 | *Trichoderma virens* | 90% | 0.49 | 0.59 |
| Scaffold 21-1 | ELIC-2 | endopolygalacturonase | AHK61120.1 | *Trichoderma virens* | 82% | 0.03 | 0.06 |
| scaffold 7-1 | ELIC-3 | Epl1 protein | CAL80753 | *Trichoderma asperellum* | 100% | 1353.02 | 693.17 |
| scaffold 6-2 | ELIC-4 | Epl2 protein | CAL80752.1 | *Trichoderma atroviride* | 88% | 0.00 | 0.00 |
| scaffold 5-2 | ELIC-5 | hydrophobin | XP_013940454.1 | *Trichoderma atroviride* | 78% | 14170.30 | 11271.70 |
| scaffold 9-1 | ELIC-6 | hydrophobin 1 | AAZ66376.1 | *Trichoderma asperellum* | 95% | 6463.64 | 3170.16 |
| scaffold 16-1 | ELIC-7 | polygalacturonase | KUF05296.1 | *Trichoderma gamsii* | 90% | 0.00 | 0.00 |
| scaffold 6-1 | ELIC-8 | swollenin | ACB05430 | *Trichoderma asperellum* | 92% | 0.50 | 1.64 |
| scaffold 11-2 | ELIC-9 | xylanase | AEM05866.1 | *Phialophora* sp. | 64% | 0.31 | 0.07 |
| scaffold 13-4 | ELIC-10 | xylanase | ANX99793.1 | *Trichoderma reesei* | 86% | 0.00 | 0.00 |
| Scaffold 21-2 | ELIC-11 | xylanase II | AFD50199.1 | *Trichoderma orientale* | 82% | 0.00 | 0.00 |
| scaffold 7-3 | ELIC-12 | endo-1,4-β-xylanase | AIG72020.1 | *Trichoderma atroviride* | 100% | 0.00 | 0.00 |
