# Supplementary material for: Identification of a novel fungus, Trichoderma asperellum GDFS1009, and comprehensive evaluation of its biocontrol efficacy
Source: PLoS One. 2017 Jun 23;12(6):e0179957. doi: 10.1371/journal.pone.0179957 (PMC5482467; doi:10.1371/journal.pone.0179957)
Supplement: S3 Table — (DOCX) [file pone.0179957.s004.docx]

**S3 Table. Antibiosis secondary metabolites analysis in spore of *T. asperellum* GDFS1009**

| **Retention time** | **Compound Name** | **Molecular Weight** |
| --- | --- | --- |
| 13.932 | Nonanal | 142 |
| 16.725 | 3,5-Dimethyloctane | 142 |
| 17.347 | 4H-Cyclopenta[3,4]Cyclobuta[1,2-B]Furan-4-One,3A,3B,5,6,6A,6B-Hexa | 150 |
| 18.537 | 3-Chloro-2-chloromethyl-1-propene | 124 |
| 19.585 | 6-Ethyl-2-methyldecane | 184 |
| 22.061 | 3E-3-Tetradecene | 196 |
| 22.259 | 5-Ethylundecane | 184 |
| 23.244 | Methyl 9-oxononanoate | 186 |
| 23.878 | 6-Pentyl-2H-pyran-2-one | 166 |
| 24.606 | 4E-4-Tetradecene | 196 |
| 24.791 | 2,3,5,8-Tetramethyldecane | 198 |
| 25.014 | Ethyl 9-oxononanoate | 200 |
| 26.308 | 2,3,11-Trimethyldodecane | 212 |
| 26.473 | 3-Methylpentadecane | 226 |
| 27.014 | 14E-14-Hexadecenal | 238 |
| 27.176 | 5-Propyltridecane | 226 |
| 28.223 | 1-4-Bromobutyl-2-piperidinone | 233 |
| 28.477 | 9E-9-Hexadecen-1-ol | 240 |
| 28.633 | Oxalic Acid, Allyl Nonyl Ester | 256 |
| 28.778 | 2-Hexyldecanol | 242 |
| 29.003 | 3,5,3',5'-Tetramethylbiphenyl | 210 |
| 29.284 | 14E-14-Hexadecenol | 238 |
| 29.448 | Elcosane | 282 |
| 29.701 | 2,2',5,5'-Tetramethylbiphenyl | 210 |
| 30.406 | Sulfurous acid, 2-Propyl dodecyl ester | 306 |
| 30.543 | Methyl 3-oxohexadecanoate | 284 |
| 30.814 | 9-Icosanylcyclohexane | 364 |
| 30.972 | Decyl isopropyl sulfite | 264 |
| 31.453 | 3-Eicosene | 280 |
| 31.592 | 10-Methyleicosane | 226 |
| 31.725 | 2,6,10,15-Tetramethylheptadecane | 226 |
| 32.124 | Methyl 12-methyltetradecanoate | 256 |
| 32.495 | Methyl 7E-7-hexadecenoate | 268 |
| 32.564 | Isopropyl myristate | 270 |
| 32.935 | Methyl palmitate | 270 |
| 33.402 | Butyl undecyl phthalate | 376 |
| 33.52 | 11-Tricosene | 322 |
| 33.648 | 14E-14-Hexadecenal | 238 |
| 33.846 | 1-Bromo-4-bromomethyldecane | 312 |
| 34.214 | Hexadecanoic Acid, Methyl Ester | 270 |
| 35.133 | Palmitic acid | 256 |
| 35.54 | Ethyl palmitate | 284 |
| 37.588 | Methyl stearate | 298 |
| 38.018 | 9Z-9-Octadecenoic acid | 282 |
| 38.57 | (+)-Z-13-Methyl-11-Pentadecen-1-ol Acetate | 282 |
| 38.8 | 1-Ethenyloxy-Hexadecane | 268 |
| 41.087 | Methyl 10E-10-octadecenoate | 296 |
| 41.52 | Octadecynoic Acid, Methyl Ester | 298 |
| 42.02 | 1,5-Diisopropyl-2,3-dimethylcyclohexane | 196 |
| 42.172 | Ethyl 9Z-9-octadecenoate | 310 |
| 42.663 | 11-Decyldocosane | 450 |
| 44.287 | 11-Decyltetracosane | 478 |
| 44.379 | Methyl (13Z)-13-docosenoate | 352 |
| 45.387 | Ethyl Erucate | 366 |
| 45.798 | Sulfurous acid, 2-Propyl undecyl ester | 298 |
| 47.275 | Pentatriacontane | 492 |
| 48.262 | 1-Hexyl-1-Nitrocyclohexane | 213 |
| 48.518 | 1-Hexyl-2-Nitrocyclohexane | 213 |
| 48.701 | 2-Bromo Dodecane | 233 |
| 50.08 | Teyratetracontane | 618 |
| 50.864 | Sulfurous acid, 2-Propyl dodecyl ester | 298 |
| 51.393 | Sulfurous acid, 2-Propyl Tridecyl ester | 306 |
| 51.827 | 8-3-Octyl-2-oxiranyl-1-octanol | 284 |
| 51.967 | 2-Bromooctadecanal | 346 |
| 52.179 | Hexadecanoic Acid, 2-Oxo-Methyl Ester | 284 |
| 52.673 | 1-Chloro-Heptacosane | 414 |
| 53.909 | 1,7-Dimethyl-4-1-MethylethylCyclodecane | 210 |
| 55.211 | Sulfurous acid, 2-Ethylhexyl tetradecyl ester | 278 |
