# Supplementary material for: Identification of a novel fungus, Trichoderma asperellum GDFS1009, and comprehensive evaluation of its biocontrol efficacy
Source: PLoS One. 2017 Jun 23;12(6):e0179957. doi: 10.1371/journal.pone.0179957 (PMC5482467; doi:10.1371/journal.pone.0179957)
Supplement: S4 Table — (DOCX) [file pone.0179957.s005.docx]

**S4 Table. Antibiosis secondary metabolites analysis in fermentation liquor of *T. asperellum* GDFS1009**

| **Retention time** | **Compound Name** | **Molecular Weight** |
| --- | --- | --- |
| 6.794 | Decane, 3,7-dimethyl- | 170 |
| 7.062 | 4-Piperidinone, 2,2,6,6-tetramethyl- | 155 |
| 8.351 | Tritetracontane | 605 |
| 9.565 | Dodecane, 2,6,11-trimethyl- | 212 |
| 9.832 | Oxalic acid, butyl 6-ethyloct-3-ylester | 286 |
| 10.447 | Tridecane, 5-methyl- | 198 |
| 10.581 | Tridecane, 2-methyl- | 198 |
| 11.255 | Decane, 3,8-dimethyl- | 170 |
| 11.48 | 1-Cyclohexyldimethylsilyloxy-3-methylbutane | 228 |
| 13.63 | Tetradecane, 2,6,10-trimethyl- | 240 |
| 14.55 | Heneicosane, 11-(1-ethylpropyl)- | 366 |
| 15.197 | Pentadecane, 2,6,10-trimethyl- | 254 |
| 15.432 | 10-Methylnonadecane | 282 |
| 16.566 | Phthalic acid, butyl isohexyl ester | 306 |
| 16.994 | 2-Bromotetradecane | 276 |
| 17.192 | Eicosane, 7-hexyl- | 366 |
| 17.374 | Heptadecane | 240 |
| 17.722 | 1-Octadecene | 252 |
| 18.668 | Tetratriacontane | 479 |
| 18.823 | Heptadecanenitrile | 251 |
| 19.481 | Heptacosane | 380 |
| 20.3 | Triacontane | 422 |
| 20.487 | Nonadecane, 9-methyl- | 282 |
| 20.647 | Heptadecane, 3-methyl- | 254 |
| 20.818 | Hentriacontane | 437 |
| 20.963 | Hexadecane, 2-methyl- | 240 |
| 22.899 | Hexadecane, 7,9-dimethyl- | 254 |
| 23.653 | Heptacosane, 1-chloro- | 414 |
